# Malignant psammomatous melanotic schwannoma mimicking adrenal cyst: case report

**DOI:** 10.1186/s43159-022-00189-w

**Published:** 2022-07-07

**Authors:** Aleksandar Zlatarov, Plamena Drenakova, Stefan Mihaylov, Neli Zgurova, Lilyana Petkova, Krasimir D. Ivanov

**Affiliations:** 1 Department of General and Operative Surgery, Medical University “Prof. Dr. Paraskev Stoyanov”, 1 Hristo Smirnenski Blvd, 9010 Varna, Bulgaria; 2 Department of Pediatric Surgery, Saint Marina University Hospital, Varna, Bulgaria; 3 General and Clinical Pathology/forensic Medicine and Deontology, Medical University of Varna, Varna, Bulgaria

**Keywords:** Adrenal gland, Psammomatous melanotic schwannoma, Laparoscopy

## Abstract

**Background:**

Melanotic schwannoma is a melanin producing nerve sheath tumors. Rarely, it can be associated with psammoma bodies, called psammomatous melanotic schwannoma. Psammomatous melanotic schwannomas are associated in up to 10% of the cases with Carney’s syndrome. The rarity of the lesion, which may present at different localizations create difficulty in placing a correct initial diagnosis. Definitive diagnosis is made after complete tumor excision and pathomorphological evaluation. The prognosis depends on the anatomical localization, local invasion and presence of a high mitotic index. The main pathomorphological differential diagnosis includes schwannomas and other melanin producing tumors as melanoma.

**Case presentation:**

We present a case of an 11-year-old female with cystic lesion adjacent to right adrenal gland, mimicking adrenal cyst. Ultrasound guided biopsy was undertaken due to the cystic appearance of the formation and the lack of certain diagnosis from the non-invasive diagnostic tests. No signs of cellular and nuclear atypism were observed. The diagnosis of benign endothelial cyst with spontaneous hemorrhage was suggested. The patient underwent transabdominal laparoscopic adrenalectomy en-bloc with the cyst to prevent spillage of the cyst content due to the intimate adhesion of the lesion to the adrenal gland and vena cava inferior. Pathomorphological examination revealed malignant psammomatous melanotic schwannoma. The adrenal gland was intact with no tumor infiltration. The patient was followed up on the 1st and 2nd month afterwards the surgery by MRI with no signs of local recurrence and postoperative complications.

**Conclusion:**

Psammomatous melanotic schwannoma near adrenal gland are rare and present difficulty with exact preoperative diagnosis. Complete resection should always be provided. Laparoscopic surgery is feasible if radical excision is not compromised. Long-term follow-up and Carney’s syndrome surveillance after complete excision are recommended especially in young patients.

## Background

Melanotic schwannoma (MS) is a melanin-producing nerve sheath tumor [1]. Up to 50% contain psammoma bodies and are thus designated psammomatous melanotic schwannomas (PMS) [2]. PMS is a rare tumor with benign course and up to 10% are associated with malignant potential. MS lesions are thought to arise sporadically. PMS is associated in up to 10% of the cases with Carney’s syndrome [2]. It may arise from intracranial structures, posterior nerve roots of the spinal canal, and less common are seen in sympathetic chain, acoustic nerve, cerebellum, orbit, choroid plexuses, soft tissue, heart, oral cavity, esophageal wall, stomach, colon, bronchus, retroperitoneum, uterine cervix, and parotid gland [3]. It affects predominantly females and it is highly seen in the fourth decade. The surgical treatment includes radical excision. The prognosis depends on the anatomical localization, local invasion, and presence of a high mitotic index. Definitive diagnosis is made after complete tumor excision and pathomorphological evaluation. The main pathomorphological differential diagnosis of PMS includes schwannomas and other melanin producing tumors as melanoma [4]. The rarity of the lesion, which may present at different localizations create difficulty in placing a correct initial diagnosis [5].

## Case presentation

An 11-year-old female patient presented to the pediatric department with complaints of nausea, vomiting, and right lumbar pain lasting for 1 week, without previous symptoms and no history of trauma.

On physical examination, the following was noted: The weight was 82 kg and body mass index (BMI) of 27.0 kg/m^2^ (above 95th percentile on the CDC (Centers for Disease Control and Prevention) age- and sex-matched BMI growth charts), active striae and acanthosis on the inner thighs; Tanner pubertal stage 4+, with menarche starting 6 months prior the admission. The blood pressure was measured routinely and was within normal range.

The initial laboratory hormonal analysis and tumor markers aimed to identify hormonal activity by the tumor or possible malignancy (Table 1). The results did not reveal any deviation from the normal values.

**Table 1:** Laboratory results

Laboratory tests:	Measured values:		Reference range:	
Free serum cortisol	09:00 a.m.	451.94 nmol/l	09:00 a.m.	118.6–618 nmol/l
	22:00 p.m.	358.29 nmol/l	22:00 p.m.	85.3–459.6 nmol/l
Aldosterone	93.5 pmol/l supine		Supine	30–650 pmol/l
Alpha fetoprotein	0.5 ng/ml		0.6–6 ng/mL	
hCG	0.9 mIU/ml		0–5.3 mIU/ml	
Neuron specific enolase	14.5 ng/ml		10.8 ± 4.5 ng/mL	

**Fig. 1:**
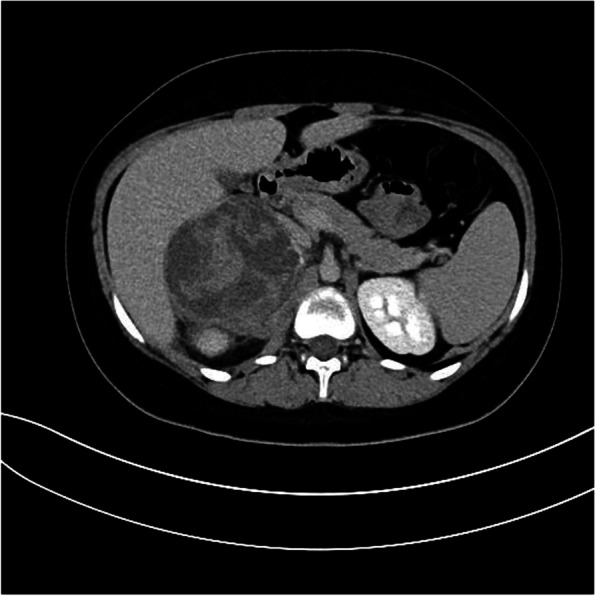
Contrast-enhanced computed tomography (CT), venous phase, axial view

The computer tomography images showed a well-defined capsulated retroperitoneal extrarenal oval lesion with heterogeneous cystic structure measuring 7.5 × 8.5 cm, native HU 5 to 95 without change after contrast enhancement (Fig. 1).

Ultrasound guided fine-needle aspiration biopsy was performed and a pig-tail drainage was placed due to the cystic appearance of the formation and the lack of certain diagnosis from the non-invasive diagnostic test. Cytological examination revealed a monomorphic population of cells with loose-textured cytoplasm and central lymphocytic-like cores with small nucleoli around a multitude of erythrocytes. No signs of cellular and nuclear atypism were observed. The drain discharged 80 ml of hemorrhagic fluid in the first 24 h and after being inactive in the next 24 h it was removed. The diagnosis of benign endothelial cyst with spontaneous hemorrhage was suggested due to imaging data suggesting a cystic lesion, the hemorrhagic characteristics of the drained fluid and the cytology analysis. The patient was observed for two days and discharged with no complains, normal vital signs and no significant change in the blood cell count. The hormonal status was to be evaluated on follow-up.

Two months later further increase in patients BMI to 30.2 kg/m^2^ was observed. Striae and inner thighs acanthosis remained. Additional hormonal laboratory tests ruled out adrenal pathology (Table 2). The elevated 24 h urine cortisol was related to obesity.

**Table 2:** Additional hormonal analysis 1 month after the initial diagnosis

Laboratory tests:	Measured values:		Reference range:	
Free serum cortisol	09:00 a.m.	451.94 nmol/l	09:00 a.m.	118.6–618 nmol/l
	22:00 p.m.	358.29 nmol/l	22:00 p.m.	85.3–459.6 nmol/l
Aldosterone	93.5 pmol/l supine		Supine	30–650 pmol/l
Alpha fetoprotein	0.5 ng/ml		0.6–6 ng/mL	
hCG	0.9 mIU/ml		0–5.3 mIU/ml	
Neuron-specific enolase	14.5 ng/ml		10.8 ± 4.5 ng/mL	

**Fig. 2:**
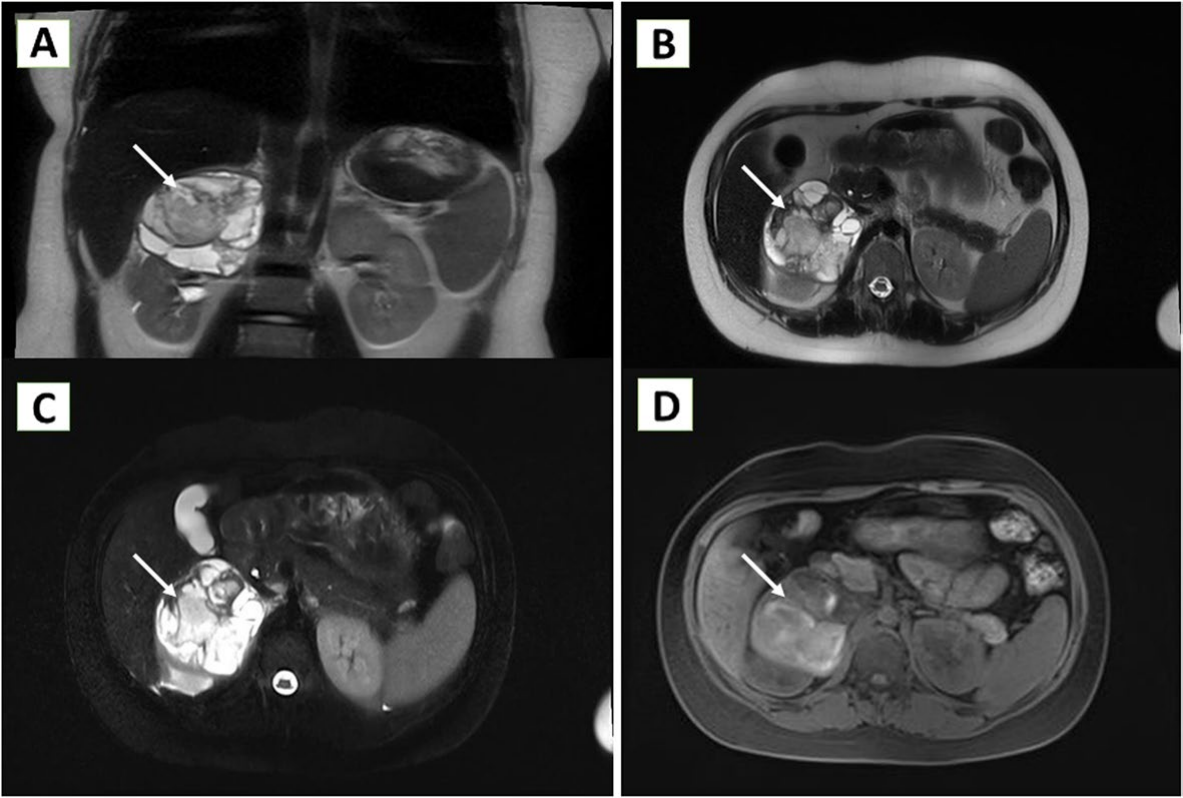
**A** T2-weighted, coronal view. **B** T2-weighted, axial view. **C** Fat suppressed sequence, axial view. **D** Gadolinium enhanced T1, axial view. The formation shows strongly heterogeneous MR-characteristic, mainly cystic (T2-hyperintense) and with hemorrhagic content (T1-hyperintense); there is presence of a relatively thick capsule (possibly a pseudocapsule due to a highly thin and peripherally dislocated parenchyma) and fine septa. After application of contrast material, there is an amplification of the signal at the periphery (capsule/pseudocapsule) of the lesion and in the septa. Mass effect on the upper pole of the right kidney, right hepatic lobe, inferior vena cava, and right renal vein is present

Abdominal ultrasound and magnetic resonance imaging (MRI) were performed and demonstrated decrease of the cyst dimensions to 52 × 50 mm (Fig. 2). The patient was scheduled for elective surgery, but due to the COVID-19 pandemics and cancellation of elective surgical procedures on national scale, the treatment was postponed.

**Fig. 3:**
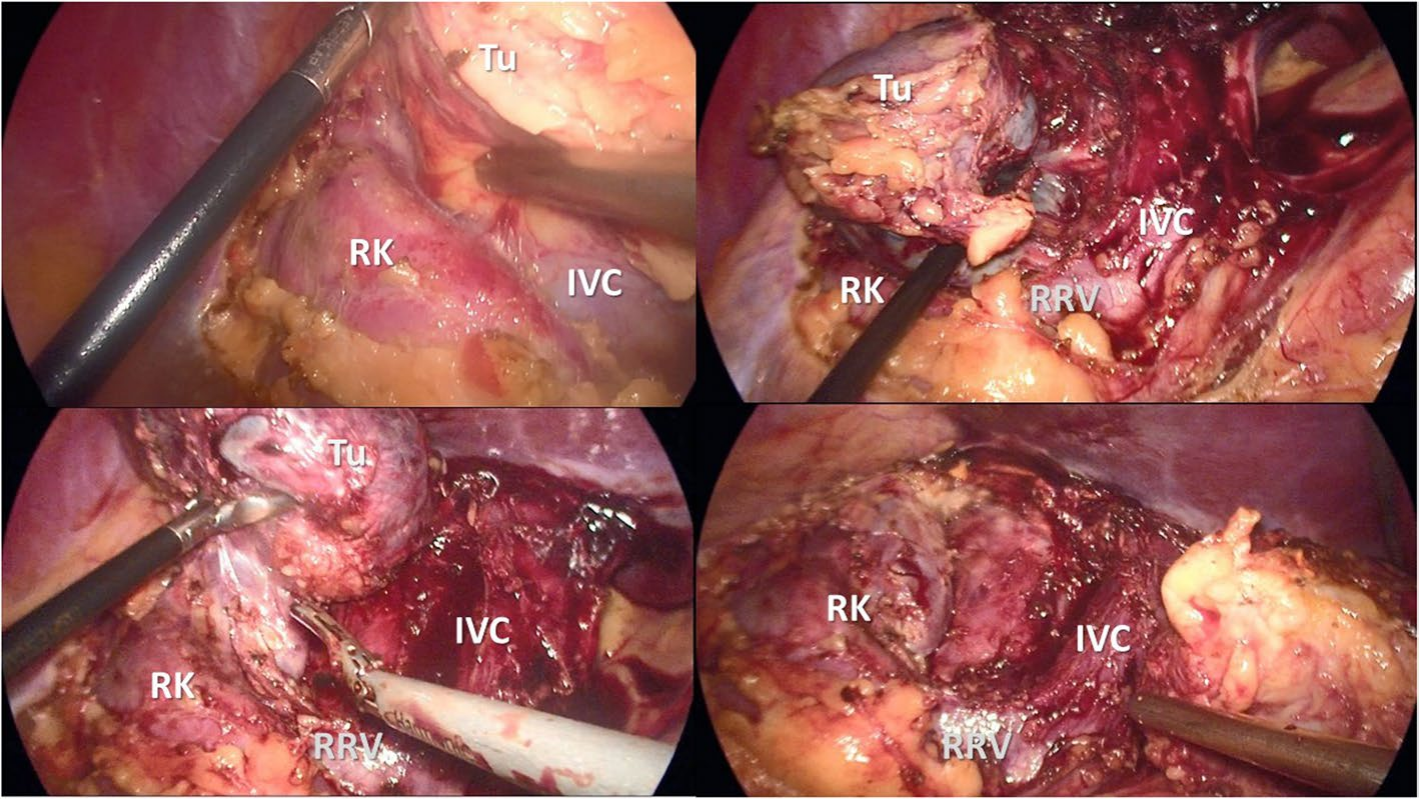
Intraoperative view of the cystic lesion; RK—right kidney; IVC—inferior vena cava; Tu—tumor; RRV—right renal vein

The patient underwent surgery 3 months after the initial hospitalization. Despite the initial plan to perform cystectomy, the intimate adhesion of the lesion to the adrenal gland and vena cava inferior necessitated en-bloc adrenalectomy to prevent spillage of the cyst content (Fig. 3). The specimen was extracted with an EndoBag.

No postoperative adverse events and complications were registered and the patient was discharged on the fourth postoperative day.

**Fig. 4:**
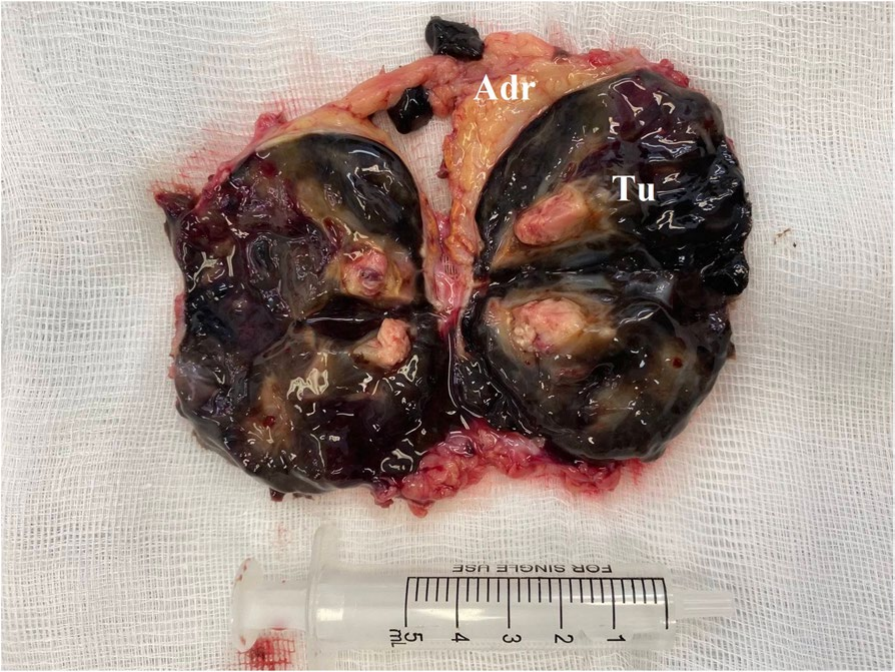
Macroscopic appearance—shining surface, intact adrenal gland—above; Adr—adrenal gland; Tu—tumor

The gross pathological description was of a lesion with dimensions 50 × 42 × 43 mm. The tumor appeared black-brown with a shining surface due to its cellular pigmentation (Fig. 4).

**Fig. 5:**
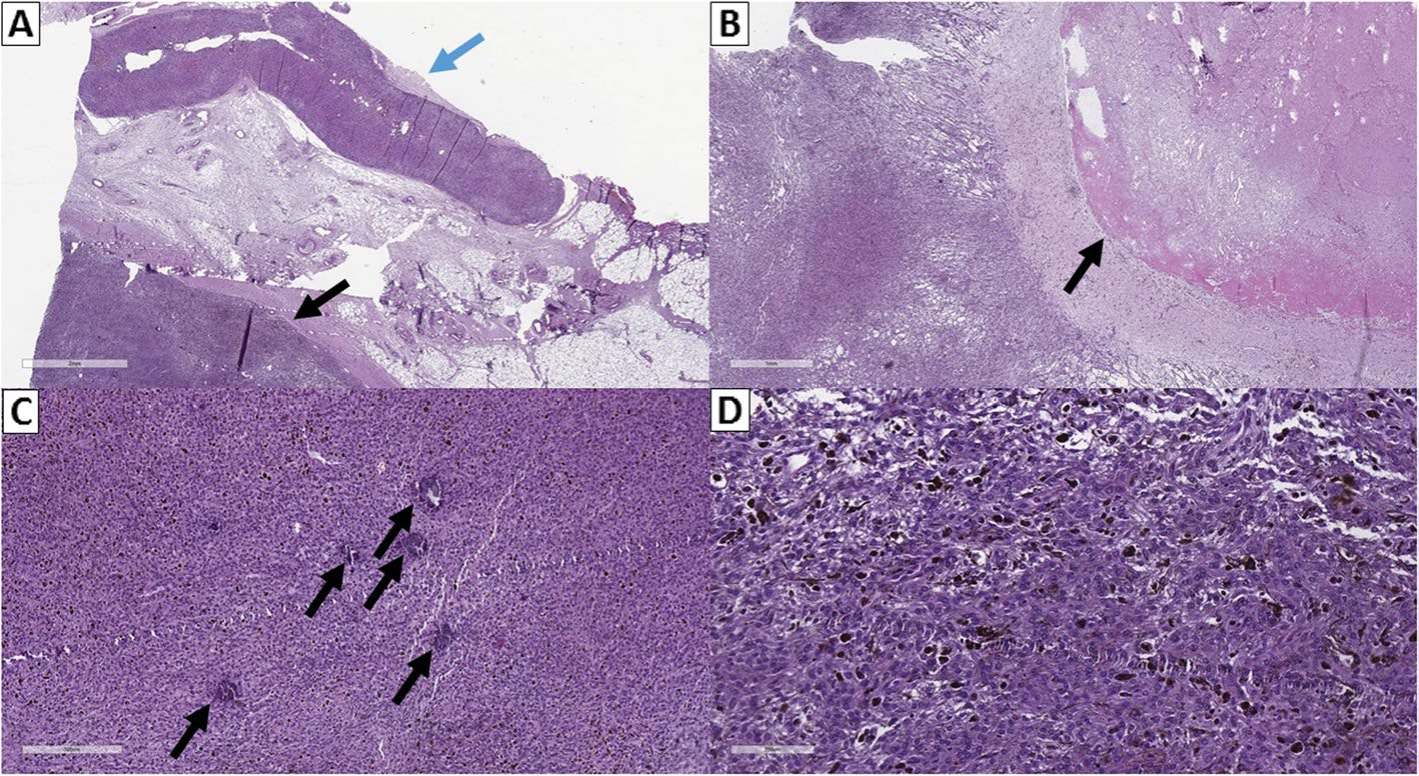
**A** Tumor formation (black arrow) without connection to the adrenal gland (blue arrow), hematoxylin and eosin stain, original magnification 14×. **B** Large field of necrosis (arrow) in the central areа of the tumor, hematoxylin and eosin stain, original magnification 20×. **C** Psammoma body calcium aggregates (arrows) within the tumor parenchyma, hematoxylin and eosin stain, original magnification 80×. **D** Hypercellular tumor parenchyma with multiple pigmented Schwan cells, hematoxylin and eosin stain, original magnification 200×

The pathomorphological description was of a tumor formation with fibrous capsule composed of drained Schwann-like cells with fasciculation, vesicular nuclei with relatively small nucleoli visualized, mitosis 6/10 high-power field (HPF), with varying amount of melanin pigment in single and groups of cells. Connective tissue stroma separated tumor cell aggregates with an abundance of psammoma bodies (calcium deposits) and foci of necrosis and hemorrhage in the tumor parenchyma. The adrenal gland was intact with no tumor infiltration (Fig. 5).

**Fig. 6:**
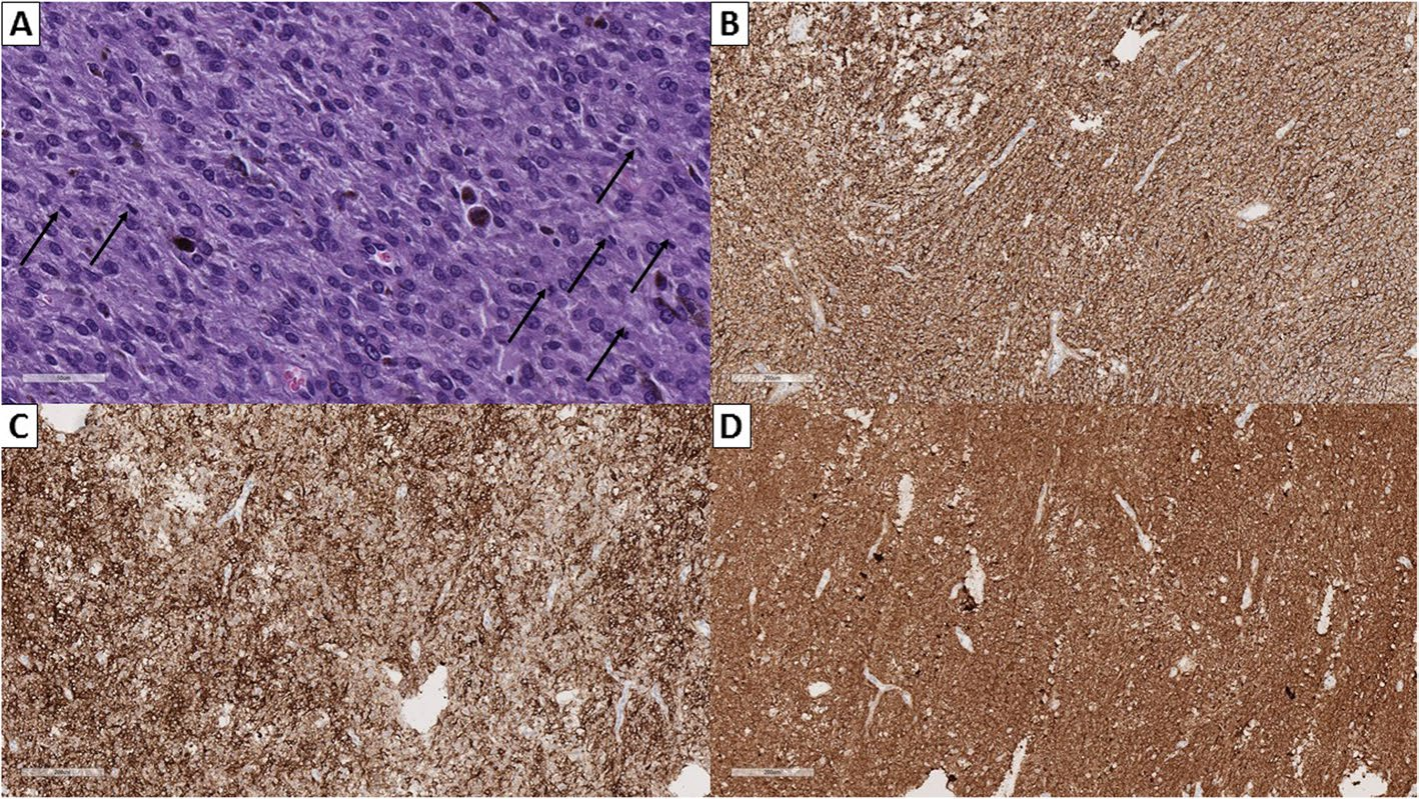
**A** Multiple mitotic figures (arrows), hematoxylin and eosin stain, original magnification 400×. **B** Strong immunopositivity for anti-HMB45, original magnification 100×. **C** Strong immunopositivity for anti-Melan A, original magnification 100×. **D** Strong immunopositivity for anti-S100, original magnification 100×

The markers S100 protein, melan-A and melanosome clone HMB45 had high-intensity expression in the tumor. Individual cells expressed inhibin alpha, Chromogranin A and Ki 67 to about 10 to 15% (Fig. 6).

The findings were conclusive of malignant psammomatous melanotic schwannoma. The main histological differential diagnosis is malignant melanoma, which shares common features such as melanin synthesis and positive staining for melanocytic markers.

The patient was followed up on the 1st and 2nd month afterwards the surgery by MRI with no signs of local recurrence and postoperative complications.

## Discussion

Melanotic schwannoma is a rare nerve sheet tumor with around 200 cases reported worldwide [6]. The first description of MS was published by Millar [7] in 1932. MS primarily occurs in intracranial structures, posterior nerve roots of the spinal canal, and less common are seen in sympathetic chain, acoustic nerve, cerebellum, orbit, choroid plexuses, soft tissue, heart, oral cavity, esophageal wall, stomach, colon, bronchus, retroperitoneum, uterine cervix, and parotid gland [3]; however, it can occur anywhere in the peripheral nervous system [6, 8, 9]. Psammomatous schwannomas near the adrenal gland have been described in less than 20 other cases [10, 11].

Female to male ratio is 1.4 and it is seen between 10 and 84 years, mostly in the fourth decade for non-psammomatous MS [3], while psammomatous melanotic schwannoma tends to occur at an earlier age, on average about 23 years [12]. The clinical course of MS is generally indolent and metastasis are rarely seen.

Establishing exact preoperative diagnosis is often challenging. Most common clinical manifestation of retroperitoneal schwannoma consists of dull flank and vague abdominal pain or uncharacterized discomfort in the affected area. Nevertheless, most of the adrenal gland tumors are diagnosed by accident due to abdominal imaging and frequently are seen larger than 4 cm in diameter. Unspecific symptoms such as digestive problems, headache, hematuria, secondary hypertension or recurrent renal colic may also be associated with retroperitoneal localization of PMS [10, 13].

In patients with retroperitoneal localization of schwannomas, the differential diagnosis is made by imaging modalities such as ultrasonography, CT or MRI. The tumors in that area such as adenoma, pheochromocytoma, myelolipoma, neurofibroma, paraganglioma, lipoma, malignant fibrous histiocytoma, and metastatic lesions must be considered [14]. The more frequent cystic lesions in that area include pseudocysts, endothelial, and epithelial cysts. Drainage of adrenal cyst could be performed in large cyst but it bears the risk of tumor seeding [15, 16].

Schwannomas have distinctive appearance on MRI–conventional (non-melanotic schwannomas) are hypointense on T1-weighted and hyperintense on T2-weighted sequences, opposite to melanotic Schwannomas which appear hyperintense on T1-weighted and hypointense on T2-weighted sequences based on the paramagnetic effects of their melanin-containing composition [3]. Khoo et al. reported no clear differentiating imaging features between the primary lesions with proven aggressive behavior on the T1- and T2-weighted, Short tau inversion recovery (STIR) or post-gadolinium enhanced conventional MRI imaging versus those that have a non-aggressive clinical course [6].

Up to date, there is no standard protocol for management due to rarity of this tumor. Surgery is the procedure of choice. When psammomatous melanotic schwannoma is suspected, biopsy of the tumor is not recommended because of risk for seeding of the tumor, hemorrhage or infection. In our case, due to uncharacteristic MRI and CT appearance, fine needle aspiration and drainage attempt was performed.

Laparoscopy is the current established approach for treating retroperitoneal and especially adrenal lesions due to lower postoperative pain and hospital stay, complication rate and comparable operative results [17]. Clinically, integration of intraoperative computer-guided navigation can decrease incidents of positive resection lines and therefore local recurrence rate [18]. Unclear resection lines can lead to local recurrence, therefore some authors advocate adjuvant radiotherapy [8, 19, 20]. In many cases, a complete resection is sufficient for the treatment of PMS or sporadic MS, but local recurrence or malignant transformation should always be kept in mind. In our patient, complete resection was performed, and therefore remain in active follow-up without adjuvant radio- or chemotherapy.

Schwannomas are neoplasms of Schwann cell origin. Although most schwannomas demonstrate classic histology, morphologic variations are occasionally encountered, such as cellular, plexiform, and melanotic schwannomas. The latter are characterized with epithelioid cells with variably sized nuclei and marked accumulation of melanin in neoplastic cells and associated melanophages. The main differential diagnosis is with other melanin-producing neoplasms, in particular melanoma. MS may occur in two forms, depending on the presence of psammoma bodies at histological examination [9]. Carney first described in 1986 psammomatous melanotic schwannomas (PMS) as part of inherited syndrome named after him [21]. PMS is widely linked to Carney’s complex which is associated with pigmented skin lesions (lentiginous or blue naevi), myxomas (in the heart, skin, and breast), multiple functional endocrine (primary pigmented nodular adrenocortical hyperplasia and pituitary) and non-endocrine (testicular) neoplasms, and unusual type of psammomatous melanotic schwannomas [4, 21]. In patients with tumors that are not linked to the Carney complex, PMS are frequently found in retroperitoneum as described in our case and posterior mediastinum. Cutaneous lesions in such cases may represent metastasis. Tumor necrosis, local invasion, and high mitotic figures are considered with metastatic potential [4]. Local recurrences and metastases are described in 35% and 44% of the cases, respectively [9].

The absence of any established post-treatment protocols underlines the necessity of long-term follow-up, especially in patients with proven aggressive histopathological findings at biopsy or resection [6]. Steins et al. reported most effective adjuvant chemotherapy with Ifosfamide and Doxorubicin in patients with melanocytic schwannoma of the neural crest and two cases of partial remission to Carboplatin and Etoposide, but the clinical value of these treatment modalities has not been well demonstrated [22].

## Conclusions

Melanotic schwannomas are rare. Exact preoperative diagnosis is difficult to achieve and they might mimic pathologies of surrounding organs or structures because of their atypical presentation. The presented patient demonstrates a rare occurrence of malignant melanotic schwannoma initially recognized as cystic formation in the adrenal gland region. Laparoscopic surgery is feasible if radical excision is not compromised. High importance is established for the proper pathomorphological differentiation between melanotic schwannomas, melanoma, and pigmented neurofibromas due to their different biological behavior and treatment protocols. Long-term follow-up and Carney’s syndrome surveillance after complete excision are recommended especially in young patients.

## Data Availability

The dataset used and analyzed during the current case report are available from the corresponding author on reasonable request.

## Abbreviations

MS: Melanotic schwannoma
PMS: Psammomatous melanotic schwannomas
CT: Computer tomography
CDC: Centers for Disease Control and Prevention
BMI: Body mass index
MRI: Magnetic resonance imaging
COVID-19: Coronavirus disease 2019
RK: Right kidney
IVC: Inferior vena cava
Tu: Tumor
RRV: Right renal vein
Adr: Adrenal gland
HPF: High-power field
HMB45: Human melanoma black 45
STIR: Short tau inversion recovery
